# Huge esophageal gastrointestinal stromal tumor successfully resected under mediastino-laparoscopic transhiatal esophagectomy: a case report

**DOI:** 10.1186/s40792-022-01464-z

**Published:** 2022-06-06

**Authors:** Keisuke Mishima, Takeshi Matsutani, Ryo Yamagiwa, Hidetsugu Hanawa, Yuji Kurihara, Norio Motoda, Nobuhiko Taniai, Hiroshi Yoshida

**Affiliations:** 1 Department of Digestive Surgery, Nippon Medical School Musashikosugi Hospital, 1-383, Kosugimachi Nakahara-ku, Kawasaki-shi, Kanagawa, 211-8533 Japan; 2 Department of Diagnostic Pathology, Nippon Medical School Musashikosugi Hospital, Kanagawa, Japan; 3 Department of Gastrointestinal Hepato-Biliary-Pancreatic Surgery, Nippon Medical School Hospital, Tokyo, Japan

**Keywords:** Esophageal gastrointestinal stromal tumor, Mediastino-laparoscopic surgery, Neoadjuvant therapy with imatinib

## Abstract

**Background:**

Esophageal gastrointestinal stromal tumors (E-GISTs) are often diagnosed early due to complaints such as dysphagia and are rarely found to be huge in size. Here, we report the treatment of a case of huge E-GIST successfully resected by minimally invasive surgery after neoadjuvant imatinib therapy.

**Case presentation:**

An 86-year-old male patient with a 3-month history of dysphagia was referred to our hospital because of a suspected mediastinal tumor on chest X-ray. The chest computed tomography scan revealed a huge solid tumor, of about 100 mm in diameter, protruding into the left thoracic cavity. Histopathological examination results of fine-needle aspiration biopsy under endoscopic ultrasonography revealed a c-kit and CD34-positive esophageal gastrointestinal stromal tumor. The patient received neoadjuvant therapy with imatinib (400 mg/day) to reduce the size of the tumor and prevent rupture during resection. After 28 days of oral administration of imatinib, the tumor size decreased. However, the patient refused to continue treatment with imatinib and therefore underwent mediastino-laparoscopic transhiatal esophagectomy. We successfully resected the tumor completely with mediastino-laparoscopic surgical techniques. Esophageal reconstruction was performed using a gastric tube in the posterior sternal route. After an uneventful postoperative course, the patient was discharged postoperative day 14. Immunohistochemical findings of the resected specimen showed that the tumor cells were positive for c-kit, DOG-1 and CD34 and negative for smooth muscle actin and S100.

**Conclusions:**

Hybrid surgical procedure utilizing mediastino-laparoscopy might be useful for high-risk patient with esophageal tumors.

## Background

Gastrointestinal stromal tumors (GISTs) compose approximately 20% of soft tissue sarcomas with an annual incidence of approximately 10 per million population [1, 2]. The most common primary sites are the stomach (60–70%) and small intestine (20–30%), followed by the colon–rectum (up to 5%) [3, 4]. However, esophageal GISTs (E-GISTs), are extremely uncommon, as they represent only 0.7% of all GIST [5, 6]. E-GISTs are often diagnosed early due to complaints such as dysphagia and are rarely found to be huge in size.

Although complete resection is the main treatment for E-GISTs, neoadjuvant therapy with tyrosine kinase inhibitors such as imatinib would be considered in the near future if preoperative diagnosis is possible with biopsy specimens [7]. In particular, we consider that neoadjuvant imatinib therapy should be evaluated for patients with huge E-GIST, with the aim of reducing tumor size, preserving surrounding organs, and reducing surgical stress. We report a rare case of huge E-GIST in an 86-year-old patient who underwent mediastino-laparoscopic transhiatal esophagectomy after neoadjuvant chemotherapy with imatinib.

## Case presentation

**Fig. 1:**
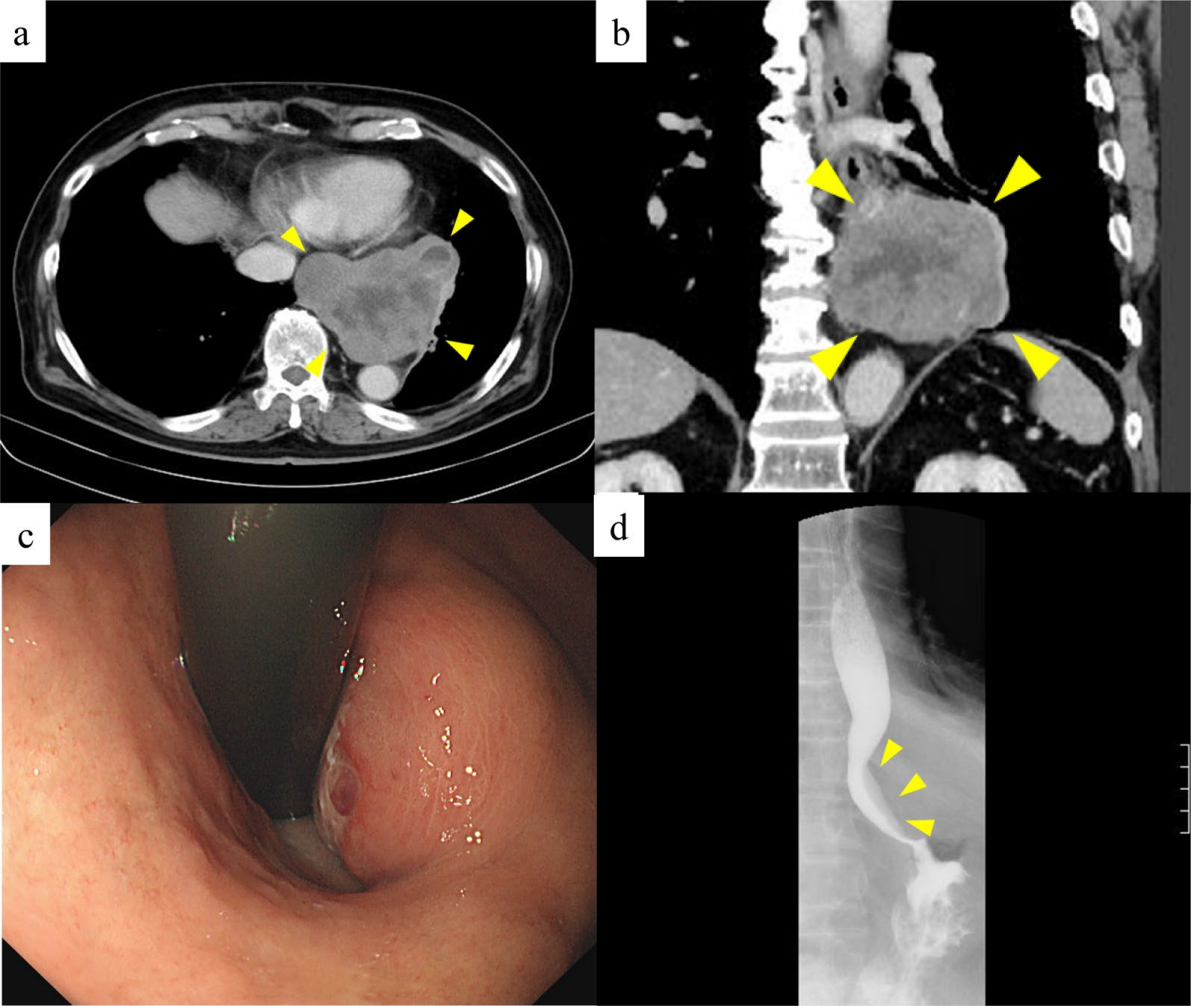
CT imaging before neoadjuvant therapy with imatinib shows that the tumor size is about 100 mm in diameter (**a**, **b**). Esophagogastroduodenoscopy reveals a submucosal tumor with a narrowed lumen and ulceration in the lower esophagus (**c**). Upper gastrointestinal series reveals compression towards the left side of the esophagus without mucosal changes (**d**)

**Fig. 2:**
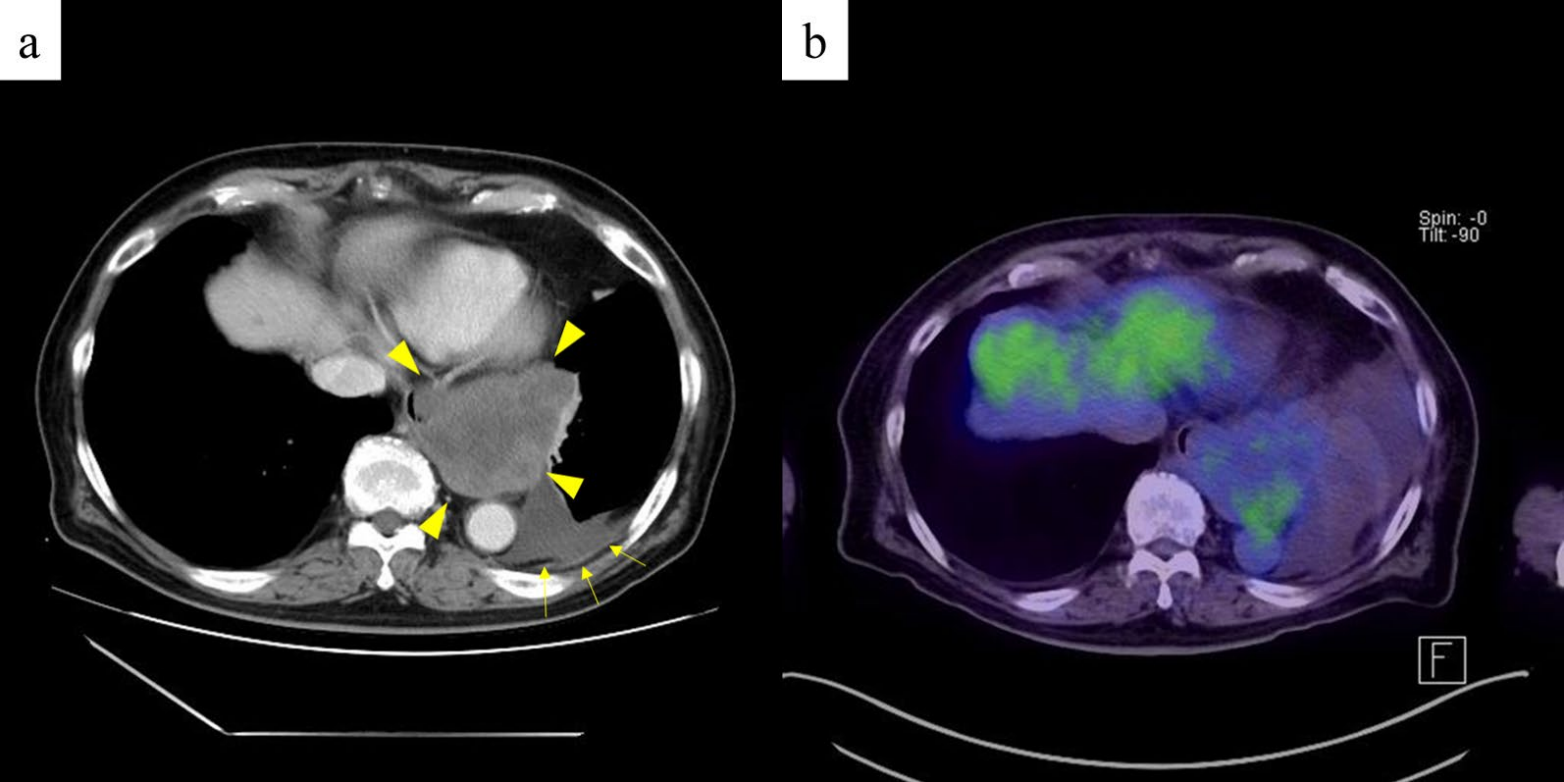
CT after neoadjuvant therapy with imatinib shows that the tumor is approximately 70 mm in diameter with pleural effusion (**a**) and PET–CT shows that SUV max was 2.6 with no apparant distant metastasis (**b**)

**Fig. 3:**
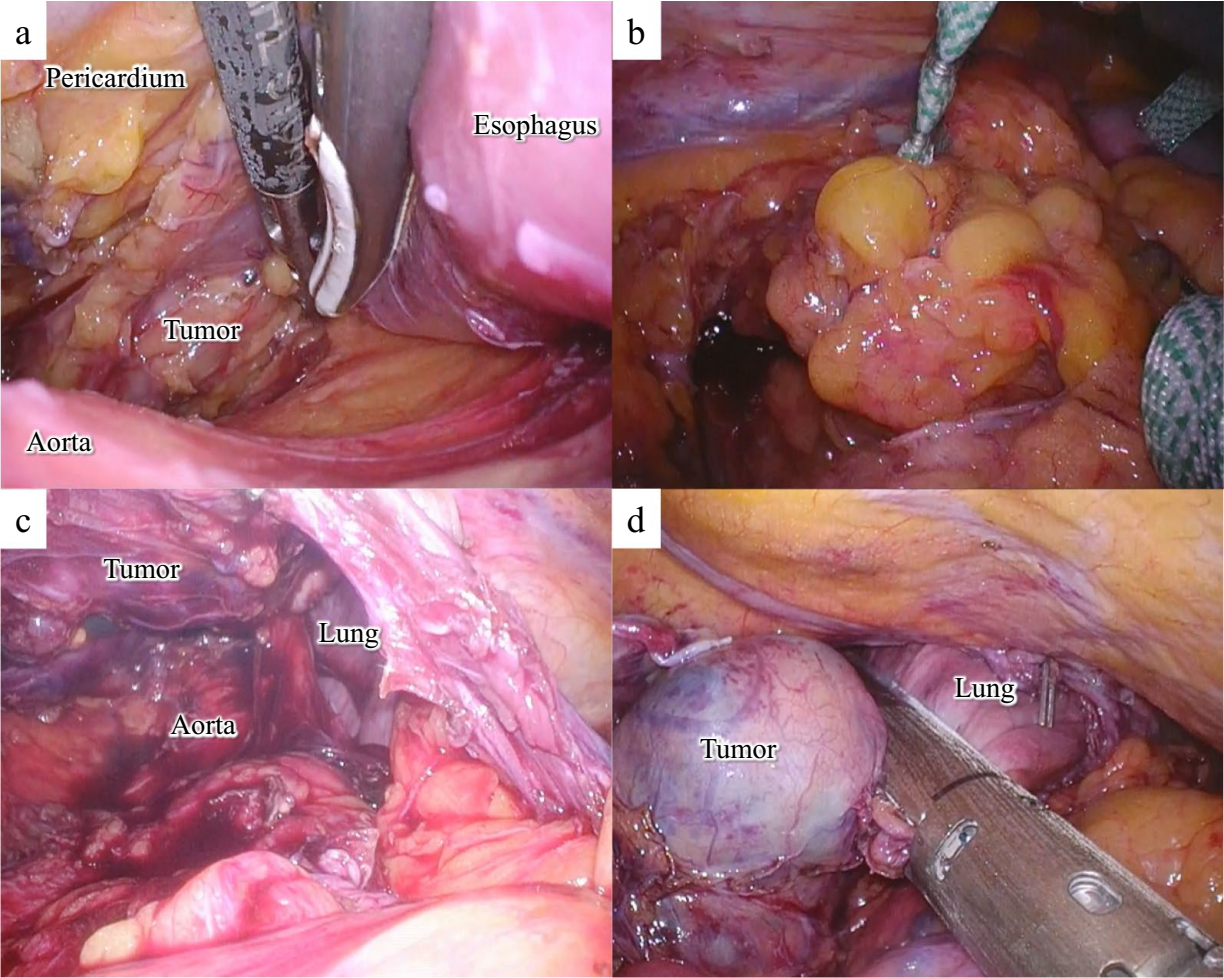
Using a mediastinoscope, the upper and middle thoracic esophagus is dissected from the surrounding tissue without lymph node dissection (**a**). A laparoscopic incision of the left diaphragm provides a clear image of the left thoracic cavity (**b**, **c**). The tumor is completely resected along with part of the lower lobe of the left lung using linear stapler (**d**)

**Fig. 4:**
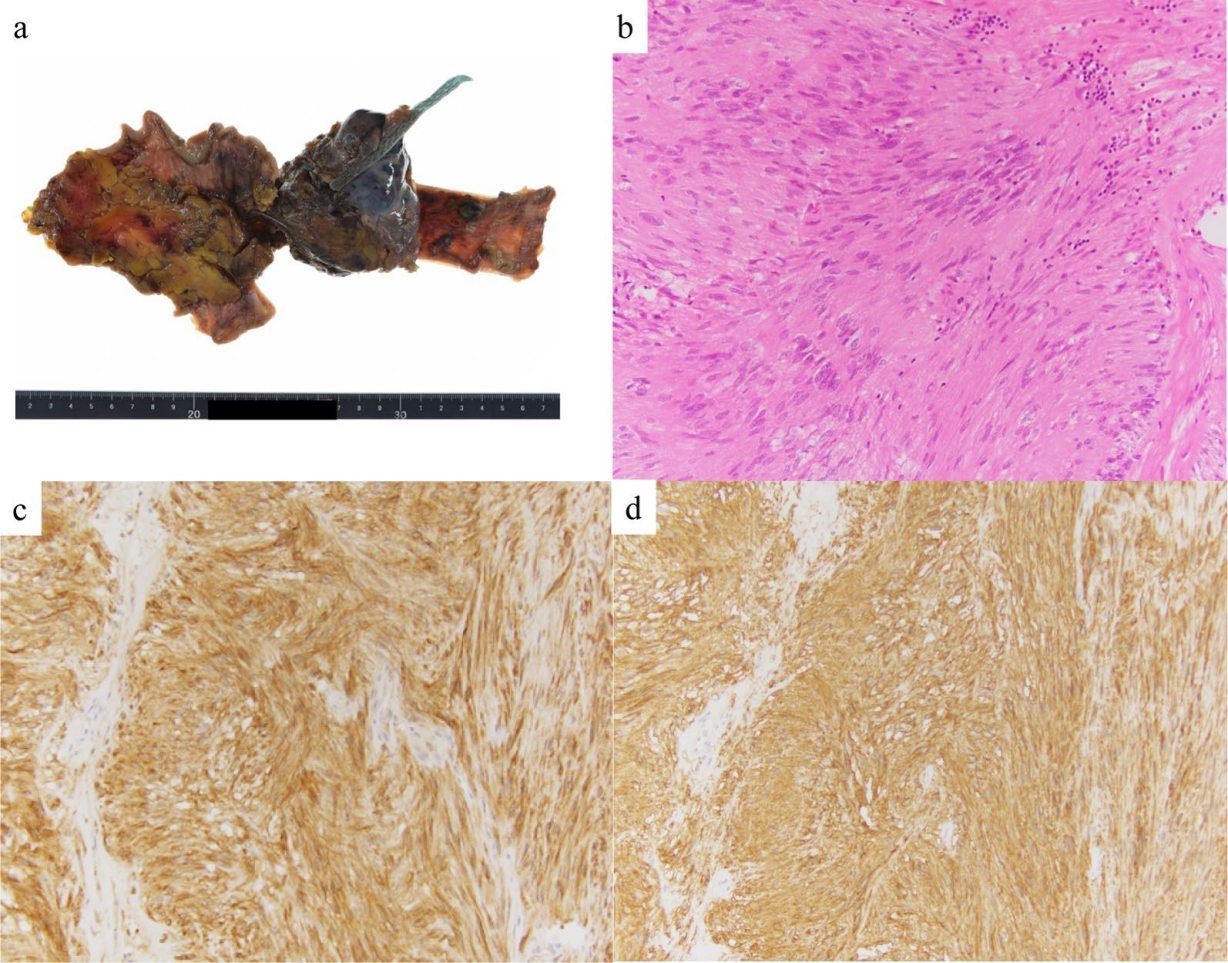
Macroscopic observation of the resected specimen shows the tumor size to be 75 × 70 × 55 mm in diameter (**a**). Histopathological findings of the tumor cells show that spindle cells densely proliferate (**b**). Immunostaining of c-kit (**c**) and DOG-1 (**d**) shows diffuse dark-brownish deposition at the cytoplasm of the spindle tumor cells

An 86-year-old Japanese male patient diagnosed with a submucosal tumor, approximately 15 mm in diameter, located in the lower esophagus 5 years ago, but had not received any treatment because of the small size of the tumor and his age of 81 years. In addition, he has a medical history of chronic obstructive pulmonary disease and was on regular health check-ups with his family doctor. The patient was referred to our hospital with dysphagia for 3 months. Physical examination and laboratory findings were normal for his age. Chest X-ray revealed a suspected mediastinal tumor. Contrast-enhanced computed tomography (CT) scan of the chest revealed a huge solid tumor, 95 × 90 × 65 mm in diameter, protruding into the left thoracic cavity (Fig. 1a, b). Esophagogastroduodenoscopy revealed a submucosal tumor with a narrowed lumen and dell in the lower esophagus (Fig. 1c), and upper gastrointestinal series revealed a smooth and round defect in the left wall of the lower-third of the esophagus (Fig. 1d). Endoscopic ultrasound showed a well-defined hypoechoic lesion on the left side of the esophagus. After a fine-needle aspiration biopsy under endoscopic ultrasonography, the tumor was diagnosed as GIST with positive immunostaining for CD34 and c-kit and negative for alpha-SMA and S100. We assessed the tumor as resectable based on these image findings, but there was a risk of tumor rupture during the surgical procedure due to the size and location of the lesion. Therefore, the patient received neoadjuvant imatinib (400 mg/day) treatment to reduce the tumor size. After 28 days of oral administration of imatinib, the size of the tumor decreased (80 × 65 × 60 mm) on chest CT scan, but a pleural effusion on the left lung field was observed (Fig. 2a). ^18^F-fluorodeoxyglucose positron emission tomography (FDG-PET) CT scan revealed increased accumulation in the tumor with a maximum standardized uptake value (SUV max) of 2.6 (Fig. 2b). There were no apparent distant metastases. The patient refused to continue the imatinib treatment because of side effects such as loss of appetite and dysphagia. Therefore, mediastino-laparoscopic transhiatal esophagectomy was performed under general anesthesia with endotracheal intubation by two-lung ventilation. An EZ access with three 5-mm ports was placed in the left cervical incision to start the pneumo-mediastinum (CO_2_ insufflation, 8 mmHg). In brief, the tissue surrounding the cervical and middle esophagus was dissected under the mediastinoscopy (Fig. 3a). Laparoscopically, traction was applied to the abdominal esophagus with cotton tape to dissect the tissue surrounding the tumor (Fig. 3b). After left thoracotomy under laparoscopy, the tumor was completely resected along with part of the lower lobe of the left lung using linear stapler (Fig. 3c, d). The left gastric artery, left gastroepiploic artery, and short gastric artery were ligated and dissected under laparoscopy, and a gastric tube was created. Esophageal reconstruction was performed using a gastric tube in the posterior sternal route. The surgical time was 415 min and the amount of blood loss was minimal. The resected tumor measured 75 × 70 × 55 mm in diameter and contained necrosis and mucosal ulceration (Fig. 4a). The microscopic findings of hematoxylin–eosin staining showed that monotonous spindle cells were densely proliferate continuing in muscularis propria of esophagus (Fig. 4b). Immunohistochemical analysis revealed that CD34, c-kit and discovered on GIST (DOG)-1 were diffusely positive in tumor cells and alfa-SMA, desmin and S-100 were negative (Fig. 4c, d). According to these results, the diagnosis of GIST was made. In addition, the tumor cells were showed as low mitotic activity (2/50 high power fields) and classified as high-risk group according to modified Fletcher classification. The postoperative course was uneventful, and the patient was discharged in good condition on postoperative day 14. The patient refused the treatment due to the side-effect of neoadjuvant imatinib despite the high risk of recurrence. There was no recurrence in the 8 months after the surgery without adjuvant imatinib administration.

## Discussion

The standard treatment for localized GISTs is complete resection of the tumor while avoiding rupture of the macroscopic and microscopic tumor capsules. Tumor rupture or the presence of residual tumor is strongly associated with recurrence and poor prognosis [8]. Although gastric and intestinal GISTs can be removed with segmental or wedge resections, the resection of E-GISTs is basically limited to enucleation or highly invasive esophagectomy due to the anatomical peculiarity of the esophagus [9]. Jiang et al. [10] recommended enucleation of E-GISTs for small tumors (2–5 cm in diameter), while esophagectomy is recommended for E-GISTs larger than 9 cm. However, the decision as to which surgical procedure should be performed for huge E-GISTs larger than 5 cm remains controversial. In our case, it was determined that a hybrid mediastinal and laparoscopic esophagectomy could resect a huge tumor approximately 8 cm in diameter. Based on the diameter and location of the tumor, we thought that there was a risk of tumor cell dissemination from intraoperative damage to the tumor capsules. Therefore, neoadjuvant treatment with imatinib (400 mg/day) was administered with the aim of reducing the tumor size.

Imatinib therapy was initially approved for advanced or metastatic GISTs and subsequently approved as adjuvant therapy after tumor resection [11]. Especially in patients with huge E-GISTs, reducing GIST size by administering imatinib preoperatively to reduce the extent of resection seems attractive, since a wide resection may result in loss of function and have a significant impact on postoperative quality of life. However, there is a little evidence based on clinical trials concerning neoadjuvant imatinib therapy for E-GISTs. Kang et al. [12] suggested that neoadjuvant imatinib therapy can be considered for patients with high mitotic rates and/or larger tumor sizes to obtain a microscopically negative margins (R0 resection) and to reduce the risk of intraoperative complications such as tumor rupture. Concerning the duration of preoperative imatinib administration, this has been reported to range from a few days to over a year [13]. The optimal duration of imatinib administration for maximum preoperative benefit is considered to be 6–12 months [14]. In our case, the duration of treatment with imatinib was not long enough, but the tumor size was reduced according to the chest CT scan.

Recently, mediastinoscopy-assisted and laparoscopic transhiatal esophagectomy with cervical anastomosis has been considered as a feasible and safe surgical procedure for selected patients with esophageal cancer [15]. In our institution, we also have performed this surgical procedure from 2017. The use of mediastinoscopy allows for clear visualization of the mediastinal structures, can be performed safely in elderly patients and high-risk cases with pulmonary disease, and may prevent postoperative pulmonary complications by avoiding the need for thoracotomy [16]; whereas, according to the Japanese GIST clinical guidelines, GISTs larger than 5 cm are not indicated for resection by minimally invasive surgery because of the risk of capsular damage. However, we believe that mediastinal and laparoscopic surgery can reduce E-GISTs capsular damage from the magnifying effect as compared to conventional open thoracotomy or laparotomy. In our patients, left and right thoracoscopic surgery was judged to be a rather difficult procedure because of the location of the huge tumor and residual esophagus, and more invasive to the patient because of the destruction of the chest wall. Therefore, the risks and benefits of minimally invasive surgery (hybrid mediastinal and laparoscopic surgery), considering their advanced age and the risk of chronic obstructive pulmonary disease, were fully explained to the patients and their families. Although a prolonged operation is needed, resection of the huge E-GIST by this hybrid mediastinal and laparoscopic procedure without the capsular rupture is possible.

A review of the literature published from January 1999 through December 2021 was performed by searching the PubMed database and the Ichushi-Web database of the Japanese Medical Abstract Society (http://login.jamas.or.jp/; NPO Japan Medical Abstracts Society) using the following key words: “esophageal gastrointestinal stromal tumor” and “surgery”. There is no report in English literature, but Japanese literature reported one case of E-GIST, 18 mm diameter, that was resected by mediastinoscope-assisted transhiatal esophagectomy (no description of laparoscopic gastric tube creation) [17]. Therefore, the present surgical report is the first case of mediastino-laparoscopic transhiatal esophagectomy for a huge GIST of more than 5 cm.

## Conclusions

We describe the treatment of a case of huge E-GIST successfully resected by minimally invasive surgery after neoadjuvant imatinib therapy. This hybrid surgical procedure utilizing mediastino-laparoscopy might be useful for high-risk patient with esophageal tumors.

## Data Availability

Not applicable.

## Abbreviations

E-GIST: Esophageal gastrointestinal stromal tumors
GISTs: Gastrointestinal stromal tumors
CT: Computed tomography
FDG-PET: Fluorodeoxyglucose positron emission tomography
SUV max: Maximum standardized uptake value
DOG: Discovered on GIST
